# Davydov Vaginoplasty in Mayer–Rokitansky–Küster–Hauser Syndrome Patient Presenting With Urethral Dilatation

**DOI:** 10.1155/2024/9498667

**Published:** 2024-06-08

**Authors:** Jurgis Vitols, Lasma Lidaka

**Affiliations:** ^1^ JV Clinic SIA “Vītols and Vītols”, Raiskuma iela 1, Riga LV-1006, Latvia; ^2^ Department of Paediatric Gynaecology Children's Clinical University Hospital, Vienibas gatve 45, Riga LV-1004, Latvia; ^3^ Department of Obstetrics and Gynaecology Rīga Stradiņš University, Riga LV-1007, Latvia

**Keywords:** Davydov vaginoplasty, Mayer–Rokitansky–Küster–Hauser syndrome, urethral dilatation, urethral intercourse

## Abstract

**Introduction:** Mayer–Rokitansky–Küster-Hauser (MRKH) syndrome is a female congenital disorder characterized by an underdeveloped or absent vagina and uterus. The first-line treatment to create a neovagina is patient-performed vaginal dilatation. We report here the rare case of an MRKH patient who presented with urethral dilatation and was successfully treated with Davydov vaginoplasty.

**Case Report:** Seventeen-year-old patient with known single kidney was consulted by a gynaecologist, and a diagnosis of MRKH syndrome was established. As the patient had urethral dilatation—resulting from repetitive intraurethral intercourse—neovaginal creation by means of self-performed vaginal dilatation was precluded. Rather, the Davydov vaginoplasty was successfully performed; there were no postoperative complications, and the patient was fully continent postsurgery.

**Conclusion:** MRKH patients and healthcare providers should be educated on the damaging consequences of intraurethral intercourse. More cases need to be reported to establish the best treatment options for a normal sexual life.

## 1. Introduction

Mayer–Rokitansky–Küster–Hauser (MRKH) syndrome is a female congenital disorder characterized by an underdeveloped or absent vagina and uterus. The prevalence of MRKH syndrome is generally considered to be around 1 in 5000 live female births. MRKH patients are divided into two types: Type I patients only exhibit agenesis of the vagina and uterus, while Type II patients also have congenital malformations of other organ systems (most often renal structural abnormalities or the absence of a kidney) [1]. Numerous genetic variations have been implicated in the development of MRKH syndrome. Genes associated with WNT signaling, homeobox genes, and other gene families crucial for female genital tract embryogenesis are believed to play pivotal roles in ensuring proper anatomical formation of the reproductive, renal, and skeletal systems. Additionally, certain environmental factors, such as endocrine-disrupting chemicals, may exert significant effects, potentially mediated through epigenetic modifications [2].

The first-line treatment for vaginal agenesis is noninvasive patient-performed vaginal dilatation [3, 4]. However, a few cases have been reported in the literature where due to different reasons (misdiagnosis, dilatation errors, etc.), dilatation or intercourse was performed in the urethra. This led to urethral dilatation or injury, consequently causing significant problems for the restoration of a normal perineal area and the formation of a functional vagina [5–7]. Several surgical techniques have been described for the development of a functional vagina. Among the most utilized are the Abbè–McIndoe technique, Williams vaginoplasty, Vecchietti vaginoplasty, Davydov vaginoplasty, and sigmoid vaginoplasty. The Abbè–McIndoe technique involves harvesting a split-thickness skin graft, typically from the buttocks, and placing it over a mould fitted within the previously created neovaginal space between the urethra and rectum. Recently other lining materials have been used (e.g., amniotic membranes, inert materials, oral mucosa, or in vitro grown vaginal tissue) [8]. In the Williams vaginoplasty, a vaginal pouch is fashioned using skin and mucosal layers from the perineum [9]. The Vecchietti and Davydov vaginoplasties are considered minimally invasive procedures. The Vecchietti procedure entails continuous upward traction on a bead positioned in the vaginal dimple, connected to sutures passing through the vesicorectal space into the abdominal cavity and through the extraperitoneal space to the anterior abdominal wall, where they are fastened to a traction device. Meanwhile, the Davydov procedure involves mobilization of a segment of the peritoneum to the introitus, subsequently allowing for spontaneous squamous epithelization of the neovagina, typically occurring over a 6-month period [10, 11]. Currently, there is no clear evidence indicating which surgical method is superior for creating a neovagina. Each technique presents potential advantages and disadvantages, and the choice often depends on individual patient factors and surgical expertise. The Davydov procedure is minimally invasive, with a short postoperative recovery period, and does not leave visible scars. It does not require tissue grafts or in vitro grown cell lines, thus precluding complications such as graft rejection or other immune reactions.

We present here the case of a patient with known single kidney who, following a gynaecology consultation, had a diagnosis of MRKH Type II established and subsequent Davydov vaginoplasty. Due to repetitive urethral intercourse, she presented with urethral dilatation and had experienced several severe pyelonephritis episodes due to recurrent ascending bacterial infections.

## 2. Case Presentation

The patient was first examined at the age of 10 years when she was admitted to the hospital due to repeated pyelonephritis episodes. Agenesis of the left kidney was detected. The patient was prepubertal at the time. Upon external genitalia examination, it was intimated that her vagina was absent and that her uterus may be absent too. Follow-up was recommended at the onset of secondary sexual characteristics; however, the patient did not seek a gynaecology consultation until the age of 17. During the first consultation, she reported that although breast development and pubic hair development had started at the age of 12, she had never menstruated. She also reported that she suffered several episodes of febrile pyelonephritis each year. The patient disclosed that she had been having regular sexual intercourse with her boyfriend for the previous 2 years; she did not experience any pain during intercourse but did observe urine leakage, which did not occur during daily or physical activities.

**Figure 1:**
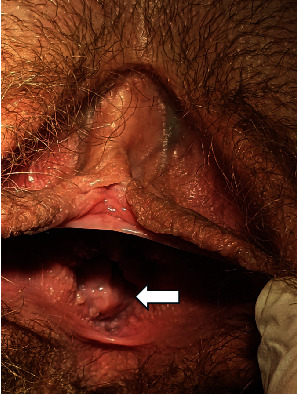
External genitalia showing normal labia majora, labia minora, and clitoris. Arrow identifies the patient's dilated urethra.

**Figure 2:**
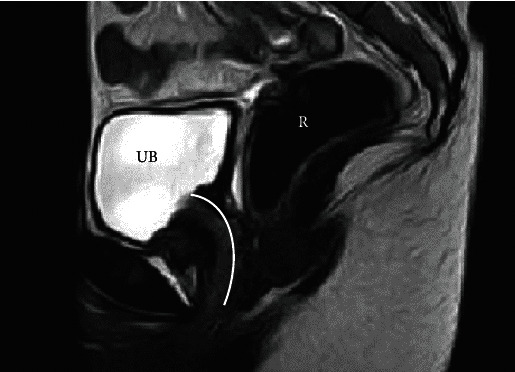
MRI. Sagittal view of pelvis, T2-weighted image, showing urinary bladder (UB), rectum (R), and dilated urethra and bladder neck (line).

On patient examination, secondary sexual characteristics corresponded to Tanner Stage V. Visual examination of external genitalia revealed normal labia majora, labia minora, and clitoris. A large orifice (approximately 2 cm in diameter) was detected between the labia minora, occupying all of the vestibule of vagina (Figure 1). Insertion of a cotton swab revealed urine leakage. Laboratory tests were performed—karyotype was 46,XX and sex hormone levels were according to pubertal stage. Transabdominal and transrectal pelvic ultrasound was performed. Preliminary diagnosis was MRKH Type II (agenesis of vagina and uterus, normal ovaries, and absent left kidney) with urethral dilatation due to repetitive urethral intercourse. Pelvic MRI examination confirmed the initial diagnosis, showing the absence of both the vagina and uterus and extensive urethral dilatation (Figure 2). Cystoscopy was performed under general anaesthesia. Hegar Uterine Dilator No. 20 was able to be freely inserted into the urethra. Cystoscopy revealed atrophic urinary bladder mucosa—a sign of chronic inflammation. Right ureteral orifice was in the correct position, and left ureteral orifice was absent as per left kidney agenesis.

The patient was informed that she should abstain from intercourse as it was likely to be one of the causes of her frequent pyelonephritis episodes. She continued to have anal sex; however, pyelonephritis episodes still occurred at least every second month. After she was advised to also abstain from anal sex, no UTIs were observed.

The restoration of a normal urethra and the creation of a neovagina posed a clinical challenge for two reasons. First, due to the patient's extensive urethral dilatation, self-performed vaginal dilatation was not possible as the dilator instantly penetrated the urethra. Second, the patient's dilated urethra occupied all of the vaginal vestibule, with only a small distance (13 mm) between the lower margin of the dilated urethra and the upper border of the anal sphincter.

The patient case was consulted via ERN eUROGEN (European Reference Network for Urogenital Diseases) using the Clinical Patient Management System (CPMS). Concerns were raised about possible injury to the urinary bladder neck and urinary bladder sphincter during surgical vaginoplasty and consequent urinary incontinence. Dorsal injury could cause anal sphincter damage and resultant flatal or faecal incontinence. Nevertheless, one colleague shared their positive experience of performing a modified Vecchietti vaginoplasty in a patient with urethral dilatation due to intraurethral coitus [12].

The patient was fully informed of all aspects of the proposed vaginoplasty. She consented to undergo the surgical procedure, with a clear understanding of the possible consequences. She disclosed that she had restarted having intercourse (apparently intraurethral) with a new partner and had already suffered several pyelonephritis episodes. She was advised to abstain from all forms of sexual activity that involved the perineal area for 6 months in an attempt to reduce the size of the urethral opening.

**Figure 3:**
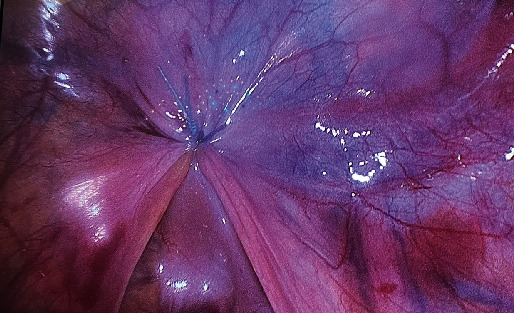
Intraperitoneal view on completion of Davydov vaginoplasty. Proximal end of the newly created vagina is closed with sutures.

**Figure 4:**
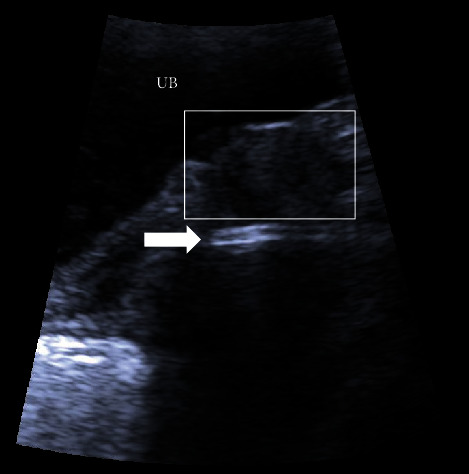
Transabdominal pelvic ultrasound, sagittal view, showing urinary bladder (UB), urethra and bladder neck (boxed area), and dilator inserted into vagina (arrow), casting a shadow. The urethra now opens at the anterior wall of the newly created vagina, and the angle precludes the possibility of accidentally penetrating it during intercourse or the dilatation process.

**Figure 5:**
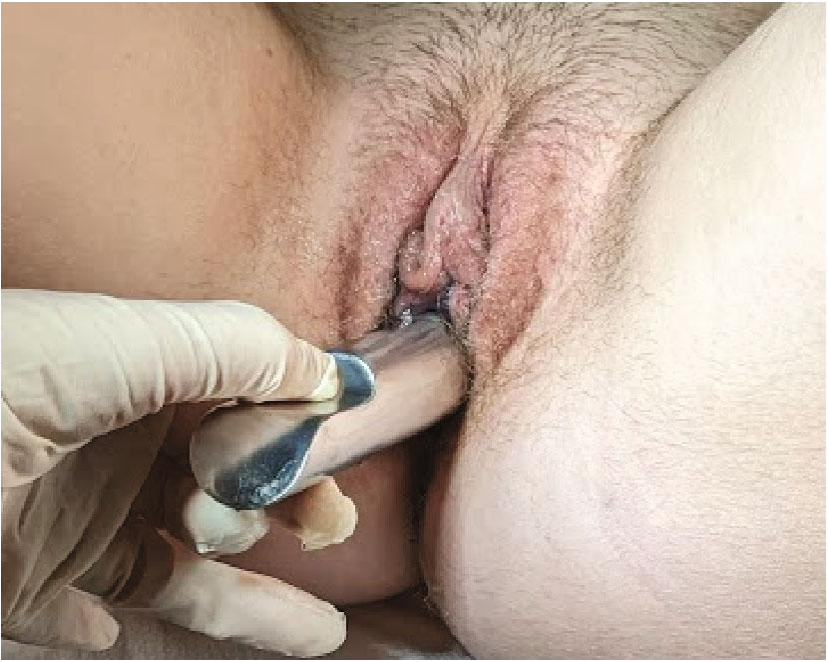
Follow-up visit, 2 months after surgery. Hegar Uterine Dilator No. 30 can be inserted approximately 8 cm deep without causing any discomfort.

Given the authors' long-standing experience with the Davydov vaginoplasty and the unavailability of a ready-made traction device typically used in the Vecchietti procedure, laparoscopic vaginoplasty, as described by Davydov, was performed (see Figure 3) [13]. The surgery proceeded remarkably smoothly. The plane of perineal dissection was meticulously controlled with visual guidance from the laparoscopy camera, and it was decided to orient it more dorsally towards the rectum. Due to the thin perineal tissue in this patient, the peritoneal cavity was reached after only a few incisions with a monopolar dissector. The later aspects of the surgery were uneventful. The mould, created from latex and filled with gauze, was inserted into the neovagina at the end of the surgery. The surgery duration was 120 min. No postsurgery complications were observed, and the mould was removed on the fourth day following the surgery. The patient was discharged on the sixth day postsurgery. As a result of the surgery, the urethral opening was pulled inwards and located at the anterior wall of the newly constructed vagina, approximately 1.5 cm from the external opening (Figure 4). The patient reported an urgency to urinate for a period of 2 months after the surgery, but this subsequently subsided. Two weeks after surgery, the patient began vaginal dilatation with a Hegar Uterine Dilator No. 20 in order to maintain and gradually enlarge her newly created vagina. During a 2-month period, dilatation was performed twice a day for 20 min; ultimately, a Hegar Uterine Dilator No. 30 was able to be inserted without any discomfort (Figure 5). At this time, she restarted intercourse with her partner and reported it to be satisfactory; no urine leakage, bowel problems, or pain were encountered. During the first year of follow-up, only one pyelonephritis episode was recorded. The patient was 19 years old at the time of publication, and she gave a written consent to publication and all pictures included.

## 3. Discussion

Intraurethral coitus in MRKH patients usually occurs when patient education/counselling has been insufficient or absent (e.g., in adult patients with primary amenorrhoea who have never been consulted by a healthcare professional regarding this issue and did not receive comprehensive sex education at school) [14–16]. Consequently, serious bladder neck injuries have been reported in some of these patients [7, 17]. Further, there have been cases where the misinterpretation of diagnostic findings or even iatrogenic injuries by healthcare providers have led to urethral and bladder neck injuries [5, 18]. Thus, these cases highlight the need for education of patients and healthcare providers regarding normal menstrual cycle parameters and normal appearance of external genitalia and also the importance of closely following patients who undergo vaginal dilatation processes.

In accordance with our MRKH patient, underreported urinary incontinence during intercourse, even if suffered for years, is a characteristic of other MRKH cases described in the literature. In line with other reports [7, 17, 19], our patient did not report this problem for 2 years. This underscores the need for men to also receive comprehensive sex education, as if they become involved in a sexual relationship where urine leakage is observed, they could be the driving force for their partner to seek medical help.

The rarity of these types of cases contributes to the different management strategies adopted by specialists treating MRKH patients. Less than 30 cases of patients who have engaged in intraurethral coitus have been published. Various techniques have been utilized in the creation of a neovagina, ranging from medically supervised self-dilatation [20] to relatively conservative surgery (Vecchietti procedure) or extensive vaginoplasty (modified McIndoe [19, 21] or sigmoid [6, 18]). The same is true for the management of a dilated urethra, with strategies such as urethral plication, sling insertion, or vaginal creation from the distal urethra simultaneously with vaginoplasty [6, 15, 16]. One major disadvantage of the classic Abbè–McIndoe method is the creation of a visible scar at the origin of the skin graft, which is often deemed unacceptable by young women. More recent techniques involve the use of buccal mucosa grafts, amniotic membranes, and in vitro cultured vaginal cells to achieve epithelialization of the neovagina. However, these methods require additional stages of tissue thawing and preparation, with the added risk of graft reactions [8, 10].

In contrast, vaginoplasty according to Davydov offers several benefits, including minimal scarring, elimination of graft harvesting, and the presence of a stratified squamous epithelium similar to that of a normal vagina. A recent systematic review comparing the Davydov and Vecchietti methods for creating a neovagina concluded that both surgeries result in comparable neovaginal length, sexual function, and complication rates. However, the Davydov surgery typically requires a significantly longer time (126 min; 95% CI 109–143) compared to the Vecchietti method (40 min; 95% CI 35–45) [11]. On the other hand, the Vecchietti method involves the utilization of a specific traction device that is attached to the abdominal wall.

As of now, there is no clear consensus regarding the best method of vaginoplasty, and decisions need to be made on a case-by-case basis, considering the expertise of the surgeon in the particular method.

We performed a relatively quick and simple Davydov vaginoplasty; no intervention was performed on the urethra. Surgery time was 120 min, that is, the same as other authors have reported [11]. The urethral opening was located at the anterior wall of the newly constructed vagina, approximately 1.5 cm from the external opening, thus precluding the possibility of accidentally penetrating it during intercourse. Our patient was fully continent after the surgery, demonstrating that it is possible to achieve good continence without additional procedures on the urethra, as previously reported [5]. On this basis, we kindly urge other colleagues to publish their cases to obtain more information regarding the best management strategies for MRKH patients.

## 4. Conclusions

We present here the case of an MRKH patient with urethral dilatation resulting from repetitive intraurethral coitus who successfully underwent Davydov vaginoplasty. The procedure was uneventful, with no postoperative complications observed. The patient had a good anatomical outcome and was fully continent after the surgery. MRKH patients and healthcare providers should be educated on the damaging consequences of intraurethral intercourse and the treatment options available to achieve a normal sexual life. Accordingly, we kindly urge other colleagues to publish more cases of MRKH patients with urethral dilatation from intraurethral coitus to gain more understanding regarding the best management strategies for these patients.

## Data Availability

The data that support the findings of this study are available from the corresponding author, LL, upon reasonable request.
